# Do EU countries comply with the right to healthcare under the UN Convention on the Rights of the Child? Cristina Hernandez Quevedo

**DOI:** 10.1093/eurpub/ckac129.331

**Published:** 2022-10-25

**Authors:** C Hernandez Quevedo, G Scarpetti, E van Ginneken

**Affiliations:** 1 London Hub, European Observatory on Health Systems and Policies, London, UK; 2 Department of Healthcare Management, Technische Universitaet Berlin, Berlin, Germany; 3 Berlin Hub, European Observatory on Health Systems and Policies, Berlin, Germany

## Abstract

Children's health status varies within and between European countries. To what extent this is associated with access barriers to timely and effective care children may face is not yet fully understood. Article 24 of the UN Convention on the Rights of the Child (UNCRC) guarantees a fundamental right to healthcare for all children, regardless of their legal status in terms of citizenship, residence, or insurance. Using information contained in the Health Systems in Transition reports produced by the European Observatory on Health Systems and Policies, additional relevant literature, and responses to a structured questionnaire filled out by key informants from all 27 EU MS and the United Kingdom, we evaluated whether European countries comply with the specific obligations that can be drawn from the UNCRC. While all countries considered have ratified the UNCRC, only four countries have included a specific disposition in their legislation that establishes an unconditional, universal right to health services for all children living in their territory. In other countries, the fragmented way of defining children's access rights can create gaps in legislation which can leave certain groups of children without coverage. Children with irregular residence are the most vulnerable group when it comes to eligibility problems, but other groups of children may also fall between the cracks or be only entitled to restrict-ed or conditional access to health care. These insights show that international treaties, such as the UNCRC, can help monitor health coverage and ensure that basic human rights to health services are guaranteed in times of crisis, such as the Covid-19 pandemic and the Ukraine Displacement, but may be insufficient without concrete transposition into national legislative frameworks.

